# (*E*)-3-[(1,5-Dimethyl-3-oxo-2-phenyl-2,3-dihydro-1*H*-pyrazol-4-yl)imino­meth­yl]phenyl 4-bromo­benzene­sulfonate

**DOI:** 10.1107/S160053680803688X

**Published:** 2008-11-13

**Authors:** Mei Li, Xin Chen

**Affiliations:** a College of Sciences, Tianjin University of Science and Technology, Tianjin 300457, People’s Republic of China

## Abstract

In the title compound, C_24_H_20_BrN_3_O_4_S, the central benzene ring makes dihedral angles of 24.55 (8), 49.52 (12) and 59.65 (7)°, respectively, with the pyrazolone ring, the bromo­benzene ring and the terminal phenyl ring. The packing is stabilized by weak non-classical inter­molecular C—H⋯O=C hydrogen bonds that form inversion-related dimers.

## Related literature

For general background to Schiff base ligands, see: Chen & Yu (2006 ▶); Kahwa *et al.* (1986 ▶); Santos *et al.* (2001 ▶); Zhao *et al.* (2006 ▶). For bond-length data, see: Allen *et al.* (1987 ▶);
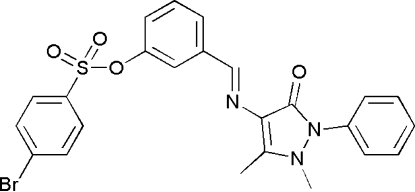


## Experimental

### 

#### Crystal data

C_24_H_20_BrN_3_O_4_S
*M* *_r_* = 526.40
Triclinic, 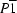

*a* = 9.3152 (17) Å
*b* = 10.1223 (18) Å
*c* = 13.472 (3) Å
α = 94.507 (3)°
β = 109.034 (3)°
γ = 102.953 (3)°
*V* = 1154.3 (4) Å^3^
*Z* = 2
Mo *K*α radiation
μ = 1.91 mm^−1^
*T* = 294 (2) K
0.20 × 0.18 × 0.10 mm

#### Data collection

Bruker SMART APEX CCD area-detector diffractometer
Absorption correction: multi-scan (*SADABS*; Sheldrick, 1996 ▶) *T*_min_ = 0.623, *T*_max_ = 0.826
6027 measured reflections
4035 independent reflections
3133 reflections with *I* > 2σ(*I*)
*R*_int_ = 0.018

#### Refinement

*R*[*F* ^2^ > 2σ(*F* ^2^)] = 0.035
*wR*(*F* ^2^) = 0.088
*S* = 1.02
4035 reflections
301 parameters
H-atom parameters constrained
Δρ_max_ = 0.27 e Å^−3^
Δρ_min_ = −0.36 e Å^−3^

### 

Data collection: *SMART* (Bruker, 1999 ▶); cell refinement: *SAINT* (Bruker, 1999 ▶); data reduction: *SAINT*; program(s) used to solve structure: *SHELXS97* (Sheldrick, 2008 ▶); program(s) used to refine structure: *SHELXL97* (Sheldrick, 2008 ▶); molecular graphics: *SHELXTL* (Sheldrick, 2008 ▶); software used to prepare material for publication: *SHELXTL*.

## Supplementary Material

Crystal structure: contains datablocks I, global. DOI: 10.1107/S160053680803688X/at2667sup1.cif

Structure factors: contains datablocks I. DOI: 10.1107/S160053680803688X/at2667Isup2.hkl

Additional supplementary materials:  crystallographic information; 3D view; checkCIF report

## Figures and Tables

**Table 1:** Hydrogen-bond geometry (Å, °)

*D*—H⋯*A*	*D*—H	H⋯*A*	*D*⋯*A*	*D*—H⋯*A*
C13—H13⋯O4	0.93	2.40	3.056 (3)	127
C21—H21⋯O4^i^	0.93	2.57	3.290 (4)	135

Symmetry code: (i) 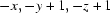.

**Fig. 1.**
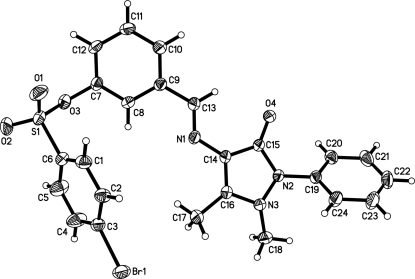
The structure of (I), with displacement ellipsoids for non-H atoms drawn at the 30% probability level.

**Fig. 2.**
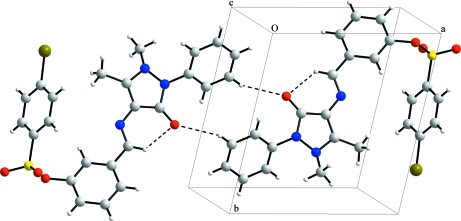
A packing diagram for (I), with hydrogen bonds drawn as dashed lines.

## supplementary crystallographic information

### Comment

Schiff-base ligands have received a good deal of attention in biology and chemistry (Kahwa *et al.*, 1986). Many Schiff base derivatives have been synthesized and employed to develop protein and enzyme mimics (Santos *et al.*, 2001). Among the large number of compounds, 4-amino-1,5-dimethyl-2-phenylpyrazol-3-one forms a variety of Schiff bases with aldehydes, and the synthesis and crystal structures of some of them, such as (*E*)-5-(1,5-Dimethyl-3-oxo-2-phenyl-2,3-dihydro- 1*H*-pyrazol-4-yliminomethyl)-2-methoxyphenyl benzenesulfonate (Chen & Yu, 2006) and (*E*)-4-(1,5-Dimethyl-3-oxo-2-phenyl-2,3-dihydro- 1*H*-pyrazol-4-yliminomethyl)-2-methoxyphenyl benzenesulfonate (Zhao *et al.*, 2006) have been reported. Structural information is useful when investigating the coordination properties of Schiff bases functioning as ligands. We report here the synthesis and molecular structure of the title Schiff base compound, (I), (Fig. 1)

In the title molecule (Fig. 1), bond lengths and angles are within normal ranges (Allen *et al.*, 1987). The pyrazolone ring (C14—C16/N1—N3/O4) is almost planar, with an r.m.s. deviation for fitted atoms of 0.0345 Å. It makes a dihedral angle of 42.12 (8)° with the attached phenyl ring (C19—C24). The central benzene ring (C7—C13/O3) is nearly planar, with an r.m.s. deviation for fitted atoms of 0.0264 Å. This group makes dihedral angles of 24.55 (8)°, 49.52 (12)° and 59.65 (7)°, respectively, with the the pyrazolone ring (C14—C16/N1—N3/O4), the bromobenzene ring (C1—C6) and the terminal phenyl ring (C19—C24).

An intramolecular C13—H13···O4═C15 hydrogen bond is found in (I) (Table 1), which helps to stabilize the conformation of the molecule. Packing is stabilized by weak, non-classical intermolecular C21—H21···O4═C15 hydrogen bonds that form inversion related dimers (Table 1, Fig. 2).

### Experimental

An anhydrous ethanol solution (50 ml) of 3-formylphenyl 4-bromobenzenesulfonate (3.41 g, 10 mmol) was added to an anhydrous ethanol solution (50 ml) of 4-amino-1,5-dimethyl-2-phenylpyrazol-3-one (2.03 g, 10 mmol) and the mixture stirred at 350 K for 3 h under N_2_, giving a yellow precipitate. The product was isolated, recrystallized from acetonitrile, and then dried in a vacuum to give pure compound (I) in 81% yield. Yellow single crystals of (I) suitable for X-ray analysis were obtained by slow evaporation of an acetonitrile solution.

### Refinement

The H atoms were included in calculated positions and refined using a riding model approximation. Constrained C—H and N—H bond lengths and isotropic U parameters: 0.93 Å and *U*_iso_(H) = 1.2*U*_eq_(C) for C*sp*^2^—H; 0.96 Å and *U*_iso_(H) = 1.5*U*_eq_(C) for methyl C—H.

### Crystal data

**Table:**

C_24_H_20_BrN_3_O_4_S	*Z* = 2
*M**_r_* = 526.40	*F*_000_ = 536
Triclinic, *P*1	*D*_x_ = 1.515 Mg m^−^^3^
Hall symbol: -P 1	Mo *K*α radiation λ = 0.71073 Å
*a* = 9.3152 (17) Å	Cell parameters from 2541 reflections
*b* = 10.1223 (18) Å	θ = 2.4–26.1º
*c* = 13.472 (3) Å	µ = 1.91 mm^−^^1^
α = 94.507 (3)º	*T* = 294 (2) K
β = 109.034 (3)º	Block, yellow
γ = 102.953 (3)º	0.20 × 0.18 × 0.10 mm
*V* = 1154.3 (4) Å^3^	

### Data collection

**Table:**

Bruker SMART APEX CCD area-detector diffractometer	4035 independent reflections
Radiation source: fine-focus sealed tube	3133 reflections with *I* > 2σ(*I*)
Monochromator: graphite	*R*_int_ = 0.018
*T* = 294(2) K	θ_max_ = 25.0º
φ and ω scans	θ_min_ = 1.6º
Absorption correction: multi-scan(SADABS; Sheldrick, 1996)	*h* = −6→11
*T*_min_ = 0.623, *T*_max_ = 0.826	*k* = −12→11
6027 measured reflections	*l* = −16→15

### Refinement

**Table:**

Refinement on *F*^2^	Hydrogen site location: inferred from neighbouring sites
Least-squares matrix: full	H-atom parameters constrained
*R*[*F*^2^ > 2σ(*F*^2^)] = 0.035	*w* = 1/[σ^2^(*F*_o_^2^) + (0.038*P*)^2^ + 0.609*P*] where *P* = (*F*_o_^2^ + 2*F*_c_^2^)/3
*wR*(*F*^2^) = 0.088	(Δ/σ)_max_ = 0.001
*S* = 1.02	Δρ_max_ = 0.27 e Å^−^^3^
4035 reflections	Δρ_min_ = −0.36 e Å^−^^3^
301 parameters	Extinction correction: SHELXL97 (Sheldrick, 2008), Fc^*^=kFc[1+0.001xFc^2^λ^3^/sin(2θ)]^-1/4^
Primary atom site location: structure-invariant direct methods	Extinction coefficient: 0.0475 (19)
Secondary atom site location: difference Fourier map	

### Special details

**Table:**

Geometry. All e.s.d.'s (except the e.s.d. in the dihedral angle between two l.s. planes) are estimated using the full covariance matrix. The cell e.s.d.'s are taken into account individually in the estimation of e.s.d.'s in distances, angles and torsion angles; correlations between e.s.d.'s in cell parameters are only used when they are defined by crystal symmetry. An approximate (isotropic) treatment of cell e.s.d.'s is used for estimating e.s.d.'s involving l.s. planes.
Refinement. Refinement of *F*^2^ against ALL reflections. The weighted *R*-factor *wR* and goodness of fit *S* are based on *F*^2^, conventional *R*-factors *R* are based on *F*, with *F* set to zero for negative *F*^2^. The threshold expression of *F*^2^ > 2σ(*F*^2^) is used only for calculating *R*-factors(gt) *etc*. and is not relevant to the choice of reflections for refinement. *R*-factors based on *F*^2^ are statistically about twice as large as those based on *F*, and *R*- factors based on ALL data will be even larger.

### Fractional atomic coordinates and isotropic or equivalent isotropic displacement parameters (Å^2^)

**Table:**

	*x*	*y*	*z*	*U*_iso_*/*U*_eq_	
Br1	1.29069 (4)	0.88146 (3)	0.88042 (3)	0.05949 (16)	
S1	1.23680 (9)	0.25329 (8)	0.93033 (8)	0.0591 (3)	
N1	0.6951 (3)	0.4506 (2)	0.71737 (18)	0.0415 (6)	
N2	0.4487 (3)	0.6559 (2)	0.57841 (19)	0.0422 (6)	
N3	0.5999 (3)	0.7481 (2)	0.61946 (19)	0.0434 (6)	
O1	1.1993 (3)	0.2290 (2)	1.02189 (19)	0.0751 (7)	
O2	1.3668 (3)	0.2167 (3)	0.9141 (3)	0.0903 (10)	
O3	1.0922 (2)	0.1687 (2)	0.82766 (17)	0.0521 (5)	
O4	0.3384 (2)	0.4347 (2)	0.59904 (17)	0.0511 (5)	
C1	1.1929 (4)	0.5050 (3)	0.9740 (2)	0.0508 (8)	
H1	1.1501	0.4679	1.0223	0.061*	
C2	1.2014 (3)	0.6396 (3)	0.9614 (2)	0.0481 (7)	
H2	1.1620	0.6935	0.9996	0.058*	
C3	1.2687 (3)	0.6936 (3)	0.8917 (2)	0.0429 (7)	
C4	1.3231 (5)	0.6147 (4)	0.8332 (3)	0.0708 (10)	
H4	1.3674	0.6525	0.7858	0.085*	
C5	1.3127 (5)	0.4803 (4)	0.8442 (3)	0.0718 (11)	
H5	1.3491	0.4262	0.8040	0.086*	
C6	1.2479 (3)	0.4251 (3)	0.9152 (2)	0.0466 (7)	
C7	0.9359 (3)	0.1566 (3)	0.8247 (2)	0.0411 (7)	
C8	0.8593 (3)	0.2500 (3)	0.7803 (2)	0.0408 (7)	
H8	0.9115	0.3239	0.7572	0.049*	
C9	0.7025 (3)	0.2331 (3)	0.7700 (2)	0.0402 (7)	
C10	0.6279 (4)	0.1203 (3)	0.8041 (2)	0.0477 (7)	
H10	0.5224	0.1068	0.7963	0.057*	
C11	0.7077 (4)	0.0283 (3)	0.8491 (2)	0.0511 (8)	
H11	0.6562	−0.0461	0.8721	0.061*	
C12	0.8633 (4)	0.0457 (3)	0.8604 (2)	0.0459 (7)	
H12	0.9181	−0.0158	0.8912	0.055*	
C13	0.6187 (3)	0.3337 (3)	0.7256 (2)	0.0423 (7)	
H13	0.5094	0.3118	0.7033	0.051*	
C14	0.6199 (3)	0.5460 (3)	0.6704 (2)	0.0386 (6)	
C15	0.4552 (3)	0.5307 (3)	0.6145 (2)	0.0380 (6)	
C16	0.7007 (3)	0.6739 (3)	0.6675 (2)	0.0414 (7)	
C17	0.8746 (3)	0.7324 (3)	0.7076 (3)	0.0617 (9)	
H17A	0.9236	0.6804	0.7589	0.093*	
H17B	0.9003	0.8264	0.7406	0.093*	
H17C	0.9121	0.7282	0.6493	0.093*	
C18	0.6390 (4)	0.8479 (3)	0.5533 (3)	0.0575 (9)	
H18A	0.7288	0.9211	0.5962	0.086*	
H18B	0.5509	0.8848	0.5229	0.086*	
H18C	0.6628	0.8035	0.4973	0.086*	
C19	0.3137 (3)	0.7097 (3)	0.5527 (2)	0.0424 (7)	
C20	0.1735 (3)	0.6289 (3)	0.4787 (2)	0.0513 (8)	
H20	0.1674	0.5420	0.4468	0.062*	
C21	0.0424 (4)	0.6802 (4)	0.4531 (3)	0.0666 (10)	
H21	−0.0533	0.6265	0.4045	0.080*	
C22	0.0526 (5)	0.8098 (5)	0.4988 (4)	0.0758 (12)	
H22	−0.0355	0.8441	0.4797	0.091*	
C23	0.1922 (5)	0.8887 (4)	0.5725 (3)	0.0729 (11)	
H23	0.1985	0.9761	0.6037	0.087*	
C24	0.3232 (4)	0.8382 (3)	0.6003 (3)	0.0575 (8)	
H24	0.4176	0.8908	0.6510	0.069*	

### Atomic displacement parameters (Å^2^)

**Table:**

	*U*^11^	*U*^22^	*U*^33^	*U*^12^	*U*^13^	*U*^23^
Br1	0.0675 (3)	0.03803 (19)	0.0694 (3)	0.00752 (14)	0.02286 (17)	0.01353 (15)
S1	0.0468 (5)	0.0447 (4)	0.0819 (6)	0.0191 (4)	0.0097 (4)	0.0255 (4)
N1	0.0401 (13)	0.0415 (13)	0.0438 (14)	0.0172 (11)	0.0109 (11)	0.0109 (11)
N2	0.0332 (12)	0.0372 (12)	0.0536 (15)	0.0124 (10)	0.0087 (11)	0.0134 (11)
N3	0.0360 (13)	0.0368 (12)	0.0551 (15)	0.0087 (10)	0.0122 (11)	0.0136 (11)
O1	0.0811 (17)	0.0595 (15)	0.0663 (16)	0.0108 (12)	0.0024 (13)	0.0320 (12)
O2	0.0494 (15)	0.0619 (16)	0.161 (3)	0.0314 (12)	0.0258 (16)	0.0295 (17)
O3	0.0504 (13)	0.0454 (12)	0.0675 (14)	0.0236 (9)	0.0218 (11)	0.0122 (10)
O4	0.0360 (11)	0.0454 (12)	0.0679 (14)	0.0091 (9)	0.0120 (10)	0.0196 (10)
C1	0.0534 (19)	0.0499 (18)	0.0507 (19)	0.0076 (14)	0.0228 (15)	0.0153 (15)
C2	0.0526 (18)	0.0402 (16)	0.0526 (19)	0.0080 (13)	0.0237 (15)	0.0040 (14)
C3	0.0439 (16)	0.0379 (15)	0.0430 (17)	0.0076 (12)	0.0123 (13)	0.0074 (13)
C4	0.103 (3)	0.055 (2)	0.082 (3)	0.024 (2)	0.063 (2)	0.0270 (19)
C5	0.101 (3)	0.056 (2)	0.092 (3)	0.037 (2)	0.064 (2)	0.022 (2)
C6	0.0444 (17)	0.0411 (16)	0.0542 (19)	0.0139 (13)	0.0136 (14)	0.0167 (14)
C7	0.0419 (16)	0.0369 (15)	0.0437 (17)	0.0155 (12)	0.0109 (13)	0.0056 (12)
C8	0.0464 (17)	0.0360 (15)	0.0417 (16)	0.0116 (12)	0.0158 (13)	0.0121 (12)
C9	0.0413 (16)	0.0382 (15)	0.0383 (16)	0.0113 (12)	0.0092 (12)	0.0082 (12)
C10	0.0425 (17)	0.0459 (17)	0.0507 (18)	0.0088 (13)	0.0127 (14)	0.0102 (14)
C11	0.057 (2)	0.0392 (16)	0.0540 (19)	0.0058 (14)	0.0182 (15)	0.0143 (14)
C12	0.0564 (19)	0.0313 (14)	0.0466 (18)	0.0143 (13)	0.0107 (14)	0.0123 (13)
C13	0.0385 (16)	0.0458 (17)	0.0433 (17)	0.0142 (13)	0.0118 (13)	0.0126 (13)
C14	0.0361 (15)	0.0405 (15)	0.0410 (16)	0.0153 (12)	0.0115 (12)	0.0107 (12)
C15	0.0372 (16)	0.0386 (15)	0.0411 (16)	0.0137 (13)	0.0137 (12)	0.0121 (12)
C16	0.0366 (15)	0.0414 (16)	0.0468 (17)	0.0143 (12)	0.0120 (13)	0.0111 (13)
C17	0.0378 (17)	0.0502 (19)	0.087 (3)	0.0096 (14)	0.0105 (17)	0.0128 (17)
C18	0.0542 (19)	0.0484 (18)	0.070 (2)	0.0110 (15)	0.0201 (17)	0.0249 (16)
C19	0.0411 (16)	0.0506 (17)	0.0445 (17)	0.0204 (13)	0.0182 (13)	0.0221 (14)
C20	0.0414 (17)	0.065 (2)	0.0489 (19)	0.0165 (15)	0.0136 (14)	0.0203 (16)
C21	0.0413 (19)	0.097 (3)	0.068 (2)	0.0250 (19)	0.0174 (17)	0.039 (2)
C22	0.062 (3)	0.102 (3)	0.098 (3)	0.054 (2)	0.042 (2)	0.058 (3)
C23	0.084 (3)	0.070 (2)	0.094 (3)	0.049 (2)	0.047 (2)	0.035 (2)
C24	0.060 (2)	0.0531 (19)	0.064 (2)	0.0269 (16)	0.0192 (17)	0.0161 (16)

### Geometric parameters (Å, °)

**Table:**

Br1—C3	1.890 (3)	C9—C10	1.390 (4)
S1—O1	1.412 (3)	C9—C13	1.467 (4)
S1—O2	1.419 (3)	C10—C11	1.376 (4)
S1—O3	1.594 (2)	C10—H10	0.9300
S1—C6	1.751 (3)	C11—C12	1.376 (4)
N1—C13	1.270 (3)	C11—H11	0.9300
N1—C14	1.390 (3)	C12—H12	0.9300
N2—C15	1.399 (3)	C13—H13	0.9300
N2—N3	1.408 (3)	C14—C16	1.355 (4)
N2—C19	1.432 (3)	C14—C15	1.437 (4)
N3—C16	1.364 (3)	C16—C17	1.488 (4)
N3—C18	1.462 (4)	C17—H17A	0.9600
O3—C7	1.420 (3)	C17—H17B	0.9600
O4—C15	1.232 (3)	C17—H17C	0.9600
C1—C2	1.375 (4)	C18—H18A	0.9600
C1—C6	1.376 (4)	C18—H18B	0.9600
C1—H1	0.9300	C18—H18C	0.9600
C2—C3	1.373 (4)	C19—C24	1.374 (4)
C2—H2	0.9300	C19—C20	1.383 (4)
C3—C4	1.363 (4)	C20—C21	1.385 (4)
C4—C5	1.366 (5)	C20—H20	0.9300
C4—H4	0.9300	C21—C22	1.374 (6)
C5—C6	1.378 (4)	C21—H21	0.9300
C5—H5	0.9300	C22—C23	1.372 (6)
C7—C8	1.368 (4)	C22—H22	0.9300
C7—C12	1.380 (4)	C23—C24	1.380 (5)
C8—C9	1.391 (4)	C23—H23	0.9300
C8—H8	0.9300	C24—H24	0.9300
			
O1—S1—O2	121.47 (17)	C10—C11—H11	119.8
O1—S1—O3	108.79 (13)	C11—C12—C7	118.3 (3)
O2—S1—O3	102.68 (16)	C11—C12—H12	120.8
O1—S1—C6	109.11 (16)	C7—C12—H12	120.8
O2—S1—C6	109.41 (15)	N1—C13—C9	120.1 (3)
O3—S1—C6	103.86 (13)	N1—C13—H13	119.9
C13—N1—C14	121.9 (2)	C9—C13—H13	119.9
C15—N2—N3	109.4 (2)	C16—C14—N1	122.1 (2)
C15—N2—C19	125.0 (2)	C16—C14—C15	108.3 (2)
N3—N2—C19	119.0 (2)	N1—C14—C15	129.5 (2)
C16—N3—N2	106.3 (2)	O4—C15—N2	123.9 (2)
C16—N3—C18	121.9 (2)	O4—C15—C14	131.5 (2)
N2—N3—C18	116.9 (2)	N2—C15—C14	104.6 (2)
C7—O3—S1	119.49 (18)	C14—C16—N3	110.7 (2)
C2—C1—C6	120.0 (3)	C14—C16—C17	127.6 (3)
C2—C1—H1	120.0	N3—C16—C17	121.7 (2)
C6—C1—H1	120.0	C16—C17—H17A	109.5
C3—C2—C1	119.1 (3)	C16—C17—H17B	109.5
C3—C2—H2	120.4	H17A—C17—H17B	109.5
C1—C2—H2	120.4	C16—C17—H17C	109.5
C4—C3—C2	121.0 (3)	H17A—C17—H17C	109.5
C4—C3—Br1	120.0 (2)	H17B—C17—H17C	109.5
C2—C3—Br1	119.0 (2)	N3—C18—H18A	109.5
C3—C4—C5	120.1 (3)	N3—C18—H18B	109.5
C3—C4—H4	120.0	H18A—C18—H18B	109.5
C5—C4—H4	120.0	N3—C18—H18C	109.5
C4—C5—C6	119.7 (3)	H18A—C18—H18C	109.5
C4—C5—H5	120.2	H18B—C18—H18C	109.5
C6—C5—H5	120.2	C24—C19—C20	120.9 (3)
C1—C6—C5	120.1 (3)	C24—C19—N2	120.9 (3)
C1—C6—S1	120.3 (2)	C20—C19—N2	118.2 (3)
C5—C6—S1	119.6 (3)	C19—C20—C21	118.6 (3)
C8—C7—C12	122.4 (3)	C19—C20—H20	120.7
C8—C7—O3	118.9 (2)	C21—C20—H20	120.7
C12—C7—O3	118.6 (2)	C22—C21—C20	120.5 (3)
C7—C8—C9	119.3 (3)	C22—C21—H21	119.7
C7—C8—H8	120.4	C20—C21—H21	119.7
C9—C8—H8	120.4	C23—C22—C21	120.3 (3)
C10—C9—C8	118.7 (3)	C23—C22—H22	119.9
C10—C9—C13	120.8 (3)	C21—C22—H22	119.9
C8—C9—C13	120.5 (2)	C22—C23—C24	119.9 (4)
C11—C10—C9	121.0 (3)	C22—C23—H23	120.1
C11—C10—H10	119.5	C24—C23—H23	120.1
C9—C10—H10	119.5	C19—C24—C23	119.8 (3)
C12—C11—C10	120.4 (3)	C19—C24—H24	120.1
C12—C11—H11	119.8	C23—C24—H24	120.1
			
C15—N2—N3—C16	8.2 (3)	O3—C7—C12—C11	−174.7 (3)
C19—N2—N3—C16	161.0 (2)	C14—N1—C13—C9	176.4 (2)
C15—N2—N3—C18	148.2 (3)	C10—C9—C13—N1	163.9 (3)
C19—N2—N3—C18	−58.9 (3)	C8—C9—C13—N1	−15.1 (4)
O1—S1—O3—C7	37.5 (2)	C13—N1—C14—C16	175.7 (3)
O2—S1—O3—C7	167.5 (2)	C13—N1—C14—C15	−7.4 (5)
C6—S1—O3—C7	−78.6 (2)	N3—N2—C15—O4	173.4 (3)
C6—C1—C2—C3	−1.6 (5)	C19—N2—C15—O4	22.5 (4)
C1—C2—C3—C4	1.7 (5)	N3—N2—C15—C14	−5.7 (3)
C1—C2—C3—Br1	−176.5 (2)	C19—N2—C15—C14	−156.6 (3)
C2—C3—C4—C5	−0.7 (6)	C16—C14—C15—O4	−177.8 (3)
Br1—C3—C4—C5	177.5 (3)	N1—C14—C15—O4	5.0 (5)
C3—C4—C5—C6	−0.4 (6)	C16—C14—C15—N2	1.2 (3)
C2—C1—C6—C5	0.5 (5)	N1—C14—C15—N2	−176.0 (3)
C2—C1—C6—S1	−179.7 (2)	N1—C14—C16—N3	−178.6 (2)
C4—C5—C6—C1	0.5 (6)	C15—C14—C16—N3	4.0 (3)
C4—C5—C6—S1	−179.2 (3)	N1—C14—C16—C17	2.4 (5)
O1—S1—C6—C1	−12.4 (3)	C15—C14—C16—C17	−175.1 (3)
O2—S1—C6—C1	−147.5 (3)	N2—N3—C16—C14	−7.5 (3)
O3—S1—C6—C1	103.5 (3)	C18—N3—C16—C14	−145.0 (3)
O1—S1—C6—C5	167.3 (3)	N2—N3—C16—C17	171.7 (3)
O2—S1—C6—C5	32.3 (3)	C18—N3—C16—C17	34.1 (4)
O3—S1—C6—C5	−76.8 (3)	C15—N2—C19—C24	124.7 (3)
S1—O3—C7—C8	93.0 (3)	N3—N2—C19—C24	−23.7 (4)
S1—O3—C7—C12	−91.3 (3)	C15—N2—C19—C20	−55.3 (4)
C12—C7—C8—C9	−0.2 (4)	N3—N2—C19—C20	156.3 (3)
O3—C7—C8—C9	175.3 (2)	C24—C19—C20—C21	0.2 (4)
C7—C8—C9—C10	−0.9 (4)	N2—C19—C20—C21	−179.8 (3)
C7—C8—C9—C13	178.2 (3)	C19—C20—C21—C22	1.3 (5)
C8—C9—C10—C11	1.3 (4)	C20—C21—C22—C23	−1.6 (5)
C13—C9—C10—C11	−177.8 (3)	C21—C22—C23—C24	0.4 (6)
C9—C10—C11—C12	−0.6 (5)	C20—C19—C24—C23	−1.3 (5)
C10—C11—C12—C7	−0.4 (4)	N2—C19—C24—C23	178.7 (3)
C8—C7—C12—C11	0.9 (4)	C22—C23—C24—C19	1.0 (5)

### Hydrogen-bond geometry (Å, °)

**Table:**

*D*—H···*A*	*D*—H	H···*A*	*D*···*A*	*D*—H···*A*
C13—H13···O4	0.93	2.40	3.056 (3)	127
C21—H21···O4^i^	0.93	2.57	3.290 (4)	135

Symmetry codes: (i) −*x*, −*y*+1, −*z*+1.
